# PAT-ChIP coupled with laser microdissection allows the study of chromatin in selected cell populations from paraffin-embedded patient samples

**DOI:** 10.1186/1756-8935-7-18

**Published:** 2014-08-05

**Authors:** Stefano Amatori, Marco Ballarini, Alice Faversani, Elena Belloni, Fulvia Fusar, Silvano Bosari, Pier Giuseppe Pelicci, Saverio Minucci, Mirco Fanelli

**Affiliations:** 1 Department of Biomolecular Sciences, University of Urbino ‘Carlo Bo’, Molecular Pathology Lab. ‘PaoLa’, Via Arco d'Augusto, 2, Fano 61032, Italy; 2 Department of Experimental Oncology, European Institute of Oncology, Via Adamello, 16, Milan 20139, Italy; 3 Division of Pathology, Fondazione IRCCS Ca' Granda Ospedale Maggiore Policlinico, Via Francesco Sforza, 33, Milan 20122, Italy; 4 Department of Pathophysiology and Transplantation, University of Milan, Via Francesco Sforza, 35, Milan 20122, Italy; 5 Department of Biosciences, University of Milan, Via Giovanni Celoria, 26, Milan 20133, Italy

**Keywords:** Chromatin immunoprecipitation, PAT-ChIP, Laser microdissection, Pathology samples, FFPE samples

## Abstract

**Background:**

The recent introduction of pathology tissue-chromatin immunoprecipitation (PAT-ChIP), a technique allowing chromatin immunoprecipitation from formalin-fixed and paraffin-embedded (FFPE) tissues, has expanded the application potential of epigenetic studies in tissue samples. However, FFPE tissue section analysis is strongly limited by tissue heterogeneity, which hinders linking the observed epigenetic events to the corresponding cellular population. Thus, ideally, to take full advantage of PAT-ChIP approaches, procedures able to increase the purity and homogeneity of cell populations from FFPE tissues are required.

**Results:**

In this study, we tested the use of both core needle biopsies (CNBs) and laser microdissection (LMD), evaluating the compatibility of these methods with the PAT-ChIP procedure. Modifications of the original protocols were introduced in order to increase reproducibility and reduce experimental time. We first demonstrated that chromatin can be prepared and effectively immunoprecipitated starting from 0.6-mm-diameter CNBs. Subsequently, in order to assess the applicability of PAT-ChIP to LMD samples, we tested the effects of hematoxylin or eosin staining on chromatin extraction and immunoprecipitation, as well as the reproducibility of our technique when using particularly low quantities of starting material. Finally, we carried out the PAT-ChIP using chromatin extracted from either normal tissue or neoplastic lesions, the latter obtained by LMD from FFPE lung sections derived from mutant K-ras^v12^ transgenic mice or from human adeno- or squamous lung carcinoma samples. Well characterized histone post-translational modifications (HPTMs), such as H3K4me3, H3K27me3, H3K27Ac, and H3K9me3, were specifically immunoselected, as well as the CTCF transcription factor and RNA polymerase II (Pol II).

**Conclusions:**

Epigenetic profiling can be performed on enriched cell populations obtained from FFPE tissue sections. The improved PAT-ChIP protocol will be used for the discovery and/or validation of novel epigenetic biomarkers in FFPE human samples.

## Background

The importance of epigenetic alterations in cancer, as well as in many other diseases, has been strongly established over the last decade. However, the epigenome and its regulation, and the mechanisms responsible for their alteration in cancer cells remain largely unknown [1-3]. To date, the majority of studies have been conducted on cultured cells; however, this approach suffers from several limitations, the most important being the appearance of molecular alterations due to the cells' adaptation to culture conditions [4,5].

In the last 20 years, chromatin immunoprecipitation (ChIP) has become a powerful experimental strategy to study the epigenome [6-10]. Total DNA obtained by ChIP is mainly analyzed at the level of single sequences by quantitative PCR (qPCR; locus-specific studies), or ‘genome-wide’ by ChIP-Seq, in order to investigate the distribution of the protein of interest over the entire genome [11,12].

These studies are producing an enormous amount of complex information that is strongly contributing to the elucidation of the epigenetic alterations involved in tumor development. Indeed, many authors believe that the time when epigenetic biomarkers (prognostic or even predictive) and/or specific epigenetic targets will start to be used in clinical practice is not far away [13,14].

Formalin-fixed paraffin-embedded (FFPE) samples are routinely used for processing and storage of pathology specimens. We have recently described the methodology, and the first application, of a new experimental procedure named pathology tissue-chromatin immunoprecipitation (PAT-ChIP), which shows that a ChIP assay can be carried out using chromatin obtained from FFPE samples [15,16]. However, a limitation of this application, common to all applications in which FFPE slides are used as starting material, is the heterogeneity of the tissue of interest. For example, tumor samples are commonly characterized by the presence of a variable amount of normal cells; this can prevent the correct identification of the cellular population contributing to a specific phenomenon. Different approaches can be employed to solve this problem. For example, tissue core needle biopsies (CNBs) directly obtained from paraffin blocks represent a good technical option to increase the purity of a cellular population. This technique has been widely exploited in the last years for applications like tissue microarrays [17,18]. After determining the region of interest, by histological staining or immunostaining of a first tissue slide, CNBs of variable diameters can be punched and recovered. The main limitation of this technique consists in the impossibility to know the precise composition of an entire CNB, as this can vary when moving from the first slide towards the inside of the paraffin block. A good alternative to this approach is represented by laser microdissection (LMD). This method exploits direct microscopic visualization to select, by laser cut, a highly enriched cellular subpopulation from the FFPE slides. Prior to excision, the slides are usually stained in order to localize the region and cellular components of interest. In addition, immunohistochemistry can be performed in those cases where antibody usage does not interfere with downstream applications [19,20]. In this study, we evaluated the possibility to apply PAT-ChIP to CNB and LMD samples.

## Results

### Application of PAT-ChIP to the study of core needle biopsies

We first evaluated the application of PAT-ChIP to CNBs obtained from FFPE samples (spleens) derived from a murine model of acute promyelocytic leukemia (APL). The spleen CNBs (0.6 mm in diameter) were fragmented by sonication (15 pulses of 15 s at 85% of amplitude): probably due to a lower specific surface than a FFPE section of equal tissue volume, a CNB needs to be sonicated by applying a higher total energy (i.e., longer total time and higher amplitude) than that normally used for a 10-μm-thick FFPE tissue section (three pulses of 30 s at 40% of amplitude). Using these conditions, we were able to isolate from CNBs an amount of chromatin comparable with that usually obtained from FFPE sections (Table 1).

**Table 1:** Fluorimetric quantification of chromatin isolated from CNBs and control FFPE sections

**Sample**	**Chromatin (μg)**
FFPE sections (n.4)	4.82
CNBs (n.4)	3.50
CNBs (n.1)	1.97

**Figure 1:**
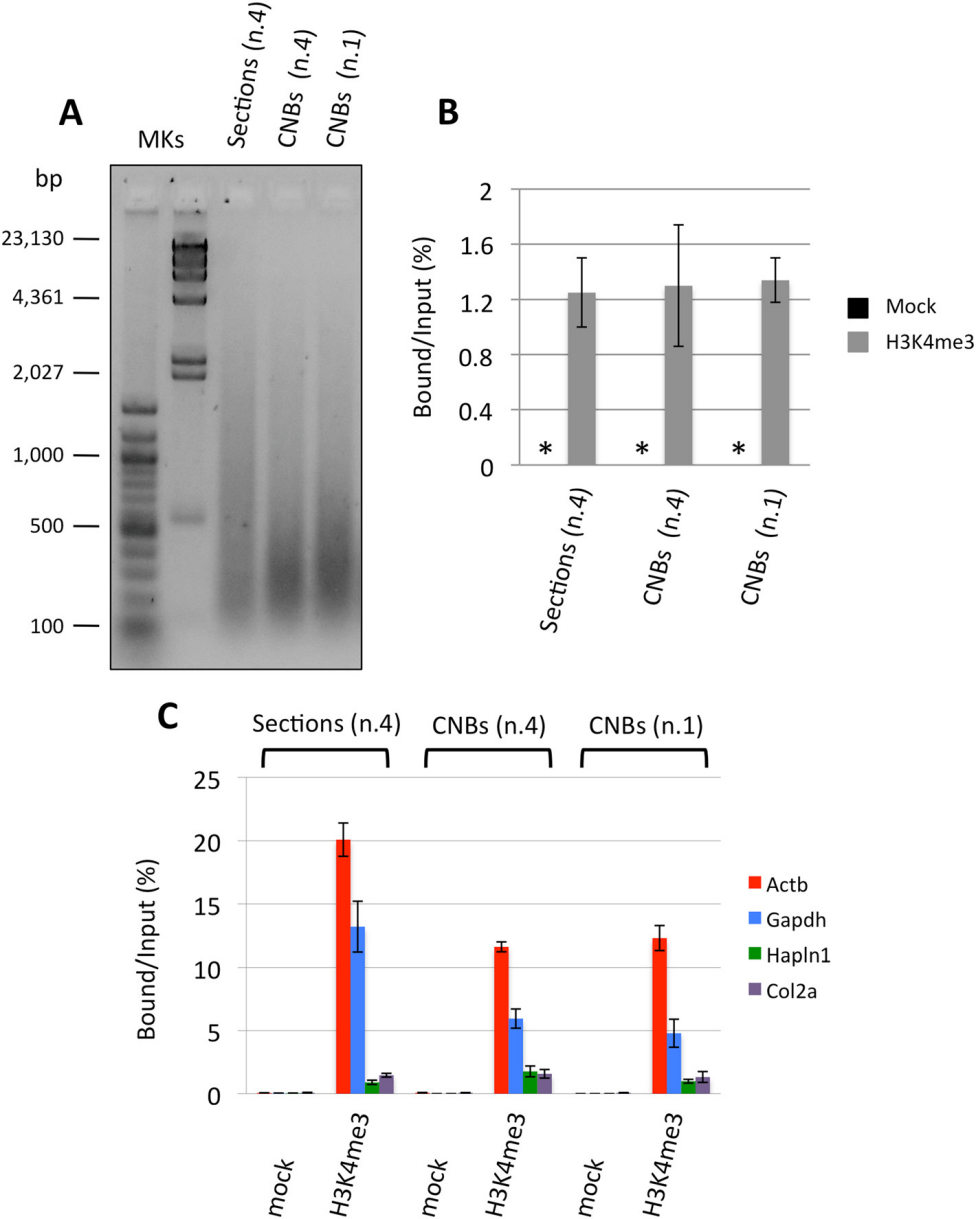
**Evaluation of the applicability of PAT-ChIP to core needle biopsies (CNBs).** Chromatin was extracted from one CNB or from a pool of four CNBs (0.6-mm diameter, 1-mm thickness) and from a pool of four FFPE tissue slides (sections, 10-μm thick) deriving from the same FFPE sample of mouse leukemic spleen. Chromatin was immunoprecipitated with an anti-H3K4me3 antibody, and the resulting purified DNA was analyzed by qPCR for enrichment at specific loci. **(A)** Evaluation of chromatin fragmentation by electrophoretic separation on 1.3% agarose gel electrophoresis (AGE) followed by ethidium bromide staining of purified input DNA. MKs, molecular weight markers. **(B)** Total amount of DNA obtained by PAT-ChIP using an anti-H3K4me3 antibody, expressed as the ratio between bound and input DNA (percentage; mean values obtained from experiment conducted in triplicate). Mock, no antibody; *, not detectable. **(C)** Amplification of transcriptionally active (Actb and Gapdh) and inactive (Hapln1 and Col2a1) promoter regions by real-time qPCR (each sample amplified in triplicate). Enrichments of the promoter sequences associated with the indicated genes for H3K4me3 (Mock, no antibody) are expressed as the bound/input ratio (percentage).

The mean fragment size obtained using CNBs was approximately 300–400 bp, whereas using FFPE sections, we obtained an average of about 500 bp: both fragments sizes are considered acceptable for what is normally required for ChIP assays (Figure 1A). Chromatin obtained from the CNBs was assayed by ChIP, taking advantage of a widely studied anti-histone H3K4me3 antibody that we had already tested for PAT-ChIP on the same FFPE spleen sections [15]. The total amount of DNA was measured, and the target DNA was expressed as the ratio of immunoprecipitated DNA relative to the input DNA, obtaining similar values for CNBs or FFPE sections (average values ranged between 1.2% and 1.4%); in contrast, no DNA was detected after ChIP assays in the absence of the antibody (mock) (Figure 1B).

The specificity of the immunoprecipitation assays was further investigated by qPCR amplifying the promoter region of four genes already used and validated in previous studies, namely, two housekeeping genes (beta-actin (Actb) and glyceraldehyde-3-phosphate dehydrogenase (Gapdh)) and two genes known to be silent in the mouse spleen (hyaluronan and proteoglycan link protein 1 (Hapln1) and collagen, type II, alpha 1 (Col2a1)) enriched or not enriched for H3K4me3 [15,16]. In all the IP samples, we found a specific and comparable enrichment of the two housekeeping genes against the silent ones and the absence of amplification in the control mock samples (Figure 1C and Additional file 1).

### Evaluation of chromatin preparation from very small tissue samples

**Figure 2:**
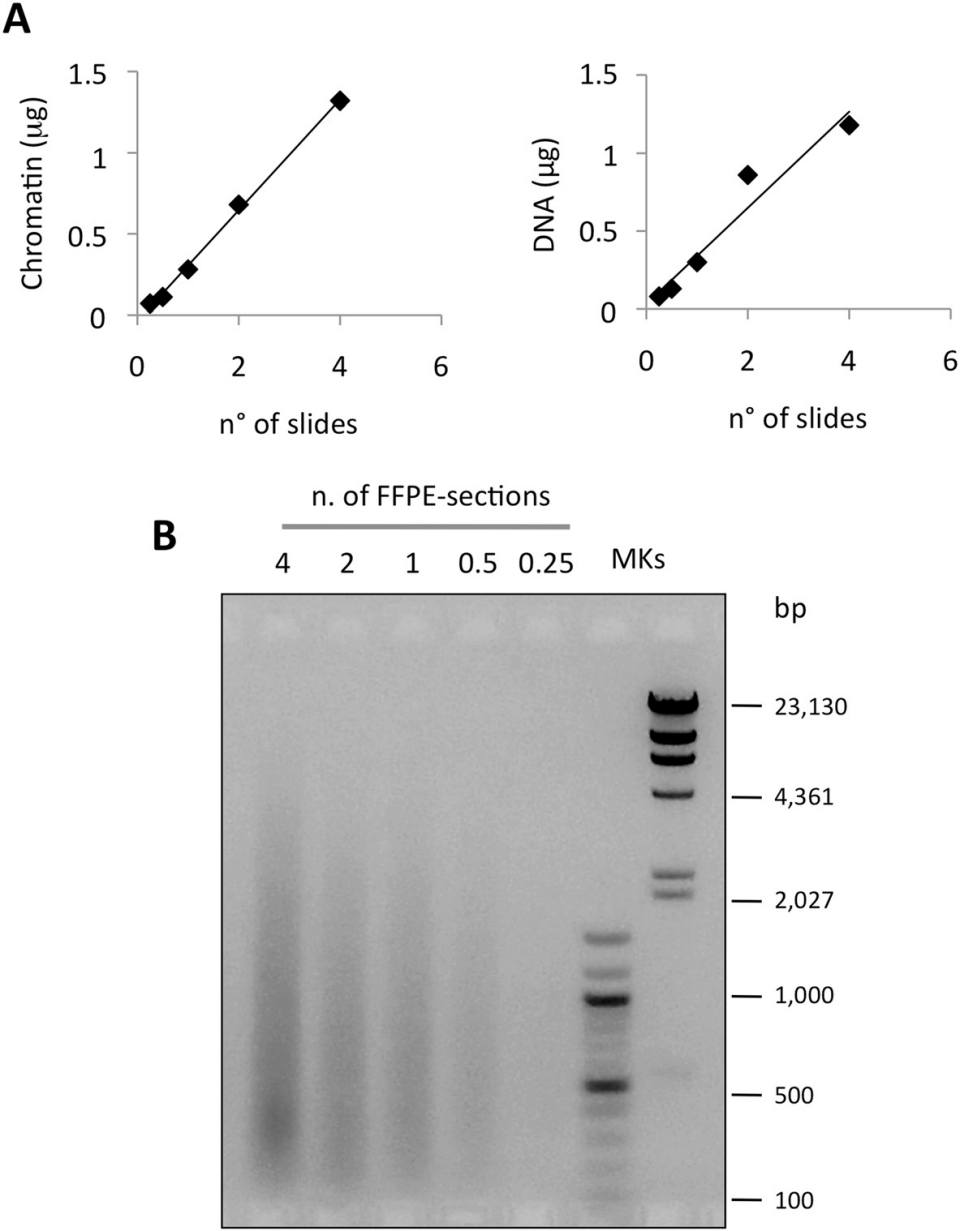
**Setting of chromatin extraction conditions for low-quantity of starting material.** After pre-fragmentation of tissue from FFPE slides taken from the lung of a 9-month-old K-ras^v12^ transgenic mouse, total tissue lysate was divided in parts equivalent to the material present in the slide number reported in the figure (4, 2, 1, 0.5, or 0.25) and subjected to chromatin extraction. **(A)** Correlation between the number of FFPE slides used as starting material and the amount of isolated chromatin before (left panel) and after (right panel) DNA purification. **(B)** Evaluation of chromatin fragmentation by electrophoretic separation on 1.3% AGE followed by ethidium bromide staining of purified DNA. MKs, molecular weight markers.

Very small tissue samples (such as those obtained by LMD) can be a very critical source of chromatin. To check the protocol for a quantitatively limited source of material and demonstrate the absence of important technical bias that might affect the procedure, we scale-down the starting material (from FFPE slides) usually used for chromatin extraction. We used the samples derived from the murine transgenic K-ras^v12^ lung carcinoma model, which, due to tissue heterogeneity, are better candidates for LMD approaches than APL samples [21]. Chromatin was extracted from murine lung tumors (FFPE slides) starting from the equivalent of 4×, 2×, 1×, 0.5×, and 0.25× FFPE lung sections. Fluorimetrical measurements of the isolated chromatin were performed prior to (chromatin) and after decrosslinking and DNA purification (DNA). As shown in Table 2, we found strong reproducibility and linearity between the quantity of starting FFPE tissues and the amount of chromatin obtained (see also Figure 2A).Chromatin fragmentation was also evaluated and, again, the different samples produced a similar fragment size distribution, with a mean around 500 bp (with the exception of the sample corresponding to the 0.25× section which appears undetectable due to the extremely low amount of material; Figure 2B).

**Table 2:** Fluorimetric quantification of chromatin isolated from different amounts of starting material

**FFPE section number**	**Isolated chromatin (μg)**	**DNA (μg)**
4	1.32	1.18
2	0.68	0.86
1	0.28	0.30
0.5	0.11	0.13
0.25	0.07	0.08

### Evaluation of the compatibility with histological staining

**Figure 3:**
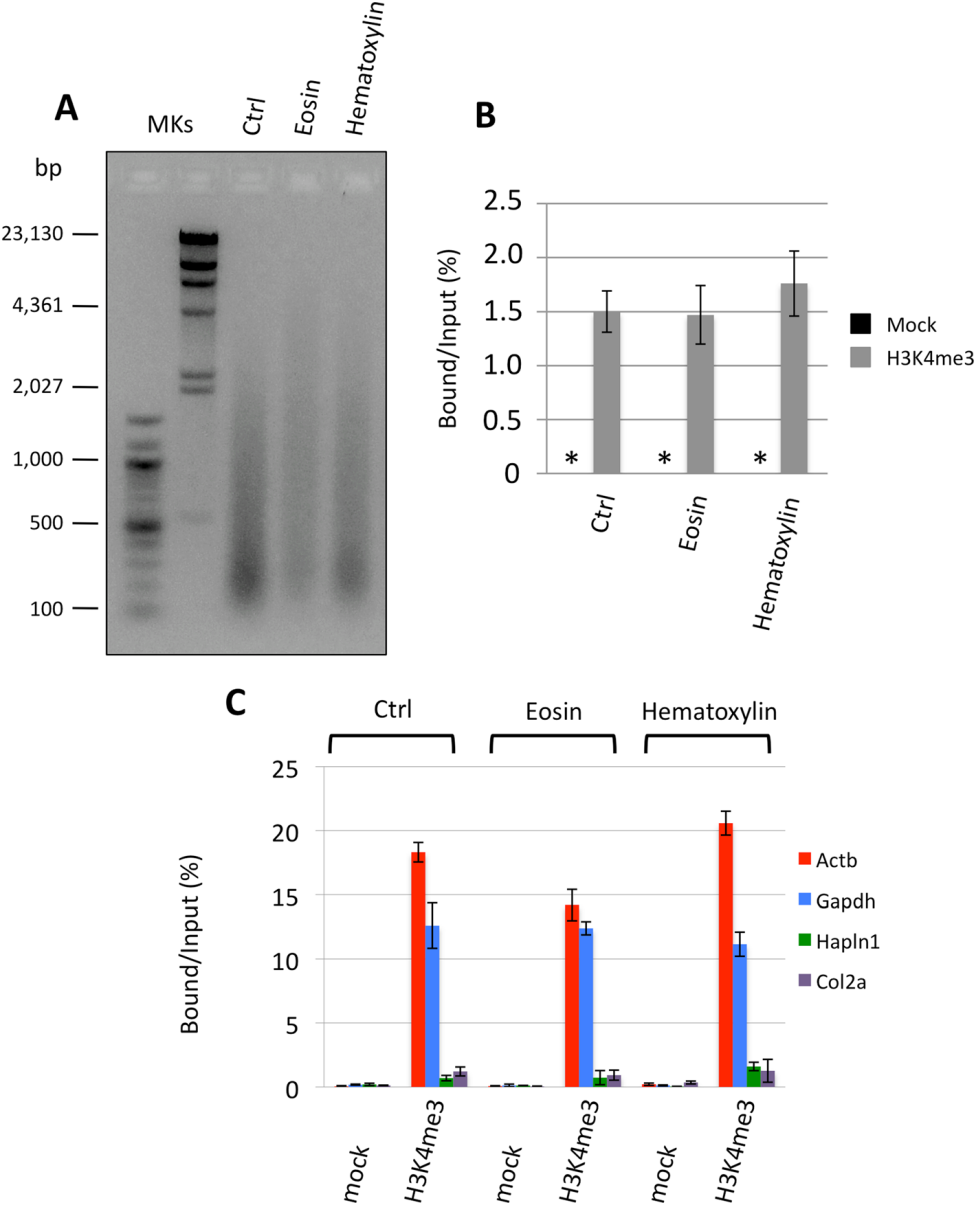
**Evaluation of the applicability of PAT-ChIP to eosin- or hematoxylin-stained tissue slides.** Chromatin was extracted from four 10-μm tissue slides stained either with eosin or hematoxylin, or not stained (Ctrl), prepared starting from the same FFPE lung sample taken from a 9-month-old K-ras^v12^ transgenic mouse. Chromatin was then immunoprecipitated with the anti-H3K4me3 antibody and the resulting purified DNA analyzed by qPCR for enrichment at specific loci. **(A)** Evaluation of chromatin fragmentation by electrophoretic separation on 1.3% AGE and by ethidium bromide staining of purified input DNA. MKs, molecular weight markers. **(B)** Total amount of DNA obtained by PAT-ChIP by using an anti-H3K4me3 antibody, expressed as the ratio between bound and input DNA (percentage; mean values obtained from experiment conducted in triplicate). Mock, no antibody; *, not detectable. **(C)** Amplification of transcriptionally active (Actb and Gapdh) and inactive (Hapln1 and Col2a1) promoter regions by real-time qPCR (each sample amplified in triplicate). Enrichments of the promoter sequences associated with the indicated genes for H3K4me3 (Mock, no antibody) are expressed as the bound/input ratio (percentage).

Tissue sections are stained with hematoxylin alone or coupled with eosin prior to laser microdissection. In order to establish if hematoxylin or eosin staining procedures could affect chromatin isolation and ChIP results, we performed parallel chromatin extractions and immunoprecipitations using four 10 μm FFPE sections, previously stained with hematoxylin or eosin (routine staining procedures) or left unstained as controls. We first evaluated the possible interference of the staining with the chromatin extraction and found no significant changes on the efficiency of chromatin isolation (Table 3 and Figure 3A).

**Table 3:** Fluorimetric estimation of the quantity of chromatin isolated from stained or unstained samples

**Sample**	**Chromatin (μg)**
Control (not stained)	1.02
Eosin	1.38
Hematoxylin	1.04

Subsequently, the chromatin was immunoprecipitated with an anti-H3K4me3 antibody, obtaining comparable results among all the samples in terms of DNA enrichment and absence of signal in mock controls (Figure 3B). The quality of the ChIP was also assessed by locus-specific qPCR, which showed a highly specific enrichment of H3K4me3 at expressed genes (Actb and Gapdh; Figure 3C and Additional file 2).

### Application of PAT-ChIP to the study of LMD samples

**Figure 4:**
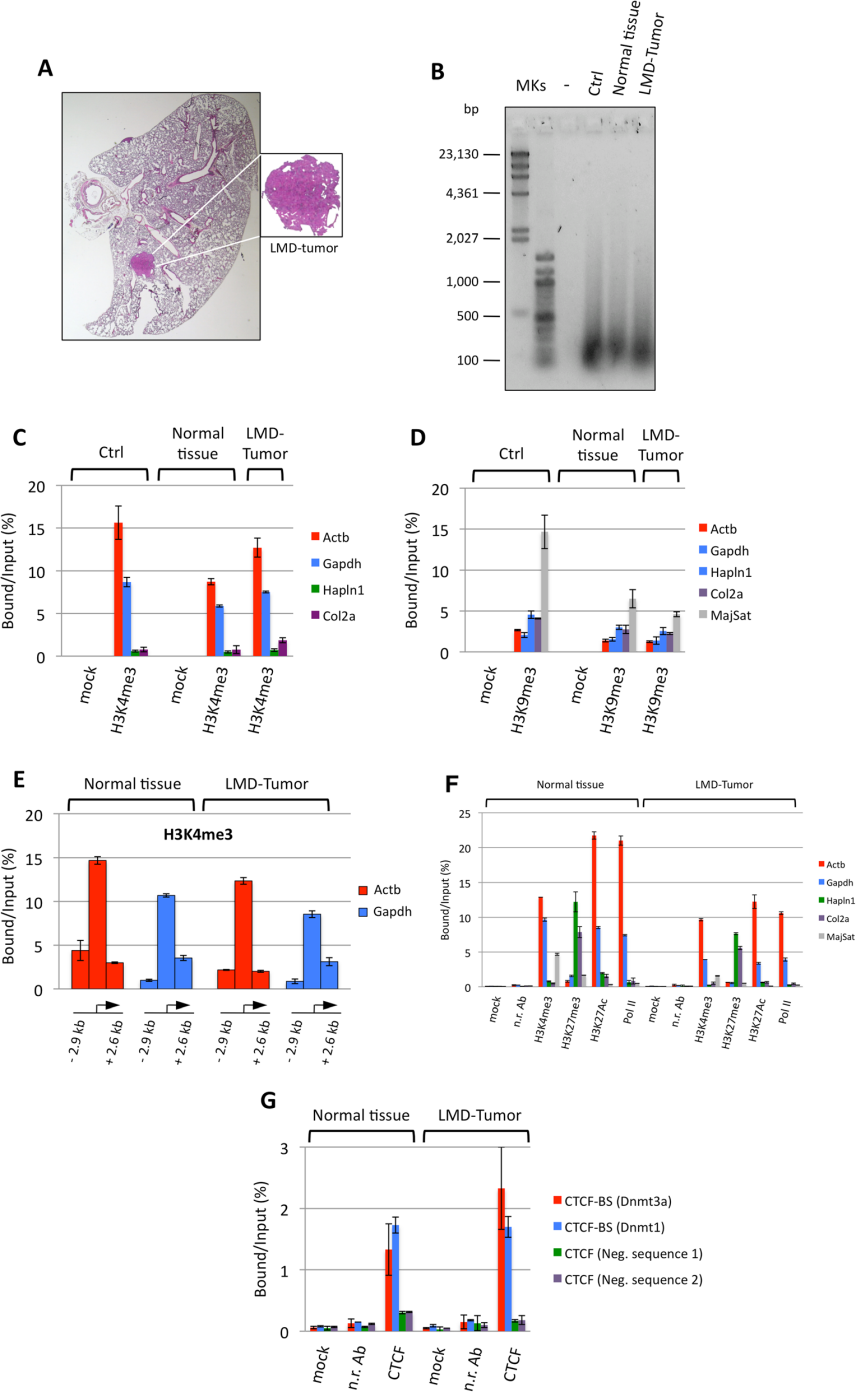
**Application of PAT-ChIP to mouse LMD samples.** Forty tissue slides were prepared from the lung of K-ras^v12^ transgenic mouse and subjected to LMD to isolate both normal and tumor cells. Chromatin was immunoprecipitated with the reported antibodies and the DNA analyzed by real-time qPCR for enrichment at specific loci (each sample amplified in triplicate). Enrichments of the amplified sequences are expressed as the ratio between bound and input (percentage). **(A)** Representative hematoxylin and eosin staining of the lung of one mice in which the expression of the oncogene was induced with 4-hydroxytamoxifen (4-OHT, right panel). **(B)** Evaluation of chromatin fragmentation by 1.3% AGE and SYBR® Gold staining of DNA purified from unbound fractions after ChIP with the H3K4me3 antibody. **(C)** Amplification of transcriptionally active (Actb and Gapdh) and inactive (Hapln1 and Col2a1) promoter regions after H3K4me3 immunselection. Mock, no antibody. **(D)** Amplification of transcriptionally active (Actb and Gapdh) and inactive (Hapln1 and Col2a1) promoter regions, and major satellite, after H3K9me3 immunoselection Mock, no antibody. **(E)** Amplification of regions located upstream and downstream from the transcription start site (TSS; at the indicated distance from TSS, see also Table 6) of the beta-actin and Gapdh genes, after H3K4me3 immunoselection. **(F)** Amplification of transcriptionally active (Actb and Gapdh) and inactive (Hapln1 and Col2a1) gene promoter regions, and major satellite sequence, after H3K4me3, H3K27me3, H3K27Ac, and Pol II immunoselections. Mock, no antibody; n.r. Ab, non-related antibody. **(G)** Amplification of two CTCF binding sites (CTCF-BS of DNA-methyltransferase 3a (Dnmt3a) and DNA-methyltransferase 1 (Dnmt1) genes) and two CTCF unrelated genomic regions as controls (CTCF neg. sequences 1 and 2), after CTCF immunoselection. Mock, no antibody; n.r. Ab, non-related antibody).

Finally, we assessed the applicability of PAT-ChIP to the LMD samples. To this end, a pilot experiment was conducted using 40 FFPE lung sections (6 μm each) derived from a K-ras^v12^ transgenic mouse; LMD was used to isolate tumor cells and normal cells from the FFPE sections (Figure 4A). Subsequently, immunoprecipitation assays, with either an anti-H3K4me3 or an anti-H3K9me3 antibody, were performed using chromatin (150 ng/assay) obtained from both normal and tumor LMD cells (as a control, a whole, not microdissected, FFPE lung section was used; Table 4). Importantly, all the chromatin samples, isolated from the different parts of the tissue sections, showed a homogeneous fragmentation, with a mean fragment size of about 300–400 bp in both microdissected samples (normal and tumor); a similar size was also found in the control chromatin (Ctrl, Figure 4B).

**Table 4:** Fluorimetric quantification of chromatin isolated from normal and LMD tumor tissue fractions

**Sample**	**Chromatin (μg)**
Normal tissue	3.56
LMD tumor	0.44

Due to the lower amount of chromatin used in comparison to the standard condition of PAT-ChIP, we decided to omit the final fluorimetrical quantitation in order to put aside sufficient material for subsequent analyses. Analysis by qPCR showed a specific immunoselection in all samples, with high levels of H3K4me3 found at the expressed genes (Actb and Gapdh), and H3K9me3 enrichment at a heterochromatic genomic region (major satellite) (Figure 4C,D and Additional file 3, panels A and B). In order to further verify the specificity of the immunoprecipitation, we analyzed the distribution of H3K4me3 in regions located upstream and downstream the transcription start sites (TSSs) of the beta-actin and Gapdh genes. In fact, as also reported in our previous study [15], H3K4me3 is a histone post-translational modification (HPTM) usually distributed in the proximity of TSSs (with the majority of peaks located in a window of ±2.5 Kb from the RefSeq annotated TSSs). As expected, genomic regions upstream and downstream the TSS of both beta-actin and Gapdh genes are less enriched in H3K4me3 than the TSS-containing region (Figure 4E).

Applicability of PAT-ChIP to LMD-FFPE samples was then further evaluated testing a panel of chromatin-related proteins (both additional HPTMs, such as H3K27me3 and H3K27Ac, and non-histone proteins, such as the transcriptional factor CTCF and RNA polymerase II), revealing specific immunoselection (Figure 4F,G and Additional file 4, panels A and B). Chromatin was isolated as described above from a further 120 FFPE lung sections (6 μm each). After immunoselection, the transcriptionally active genes were found to be enriched in H3K4me3, H3K27Ac, and Pol II PAT-ChIPs, while the repressed genes were found enriched, exclusively, in the H3K27me3 PAT-ChIP (Figure 4F and Additional file 4, panel A). Moreover, PAT-ChIP carried out using an anti-CTCF antibody showed the specific immunoprecipitation of two known CTCF binding sequences of Dnmt1 and Dnmt3a regulatory regions (Figure 4G and Additional file 4, panel B).

**Figure 5:**
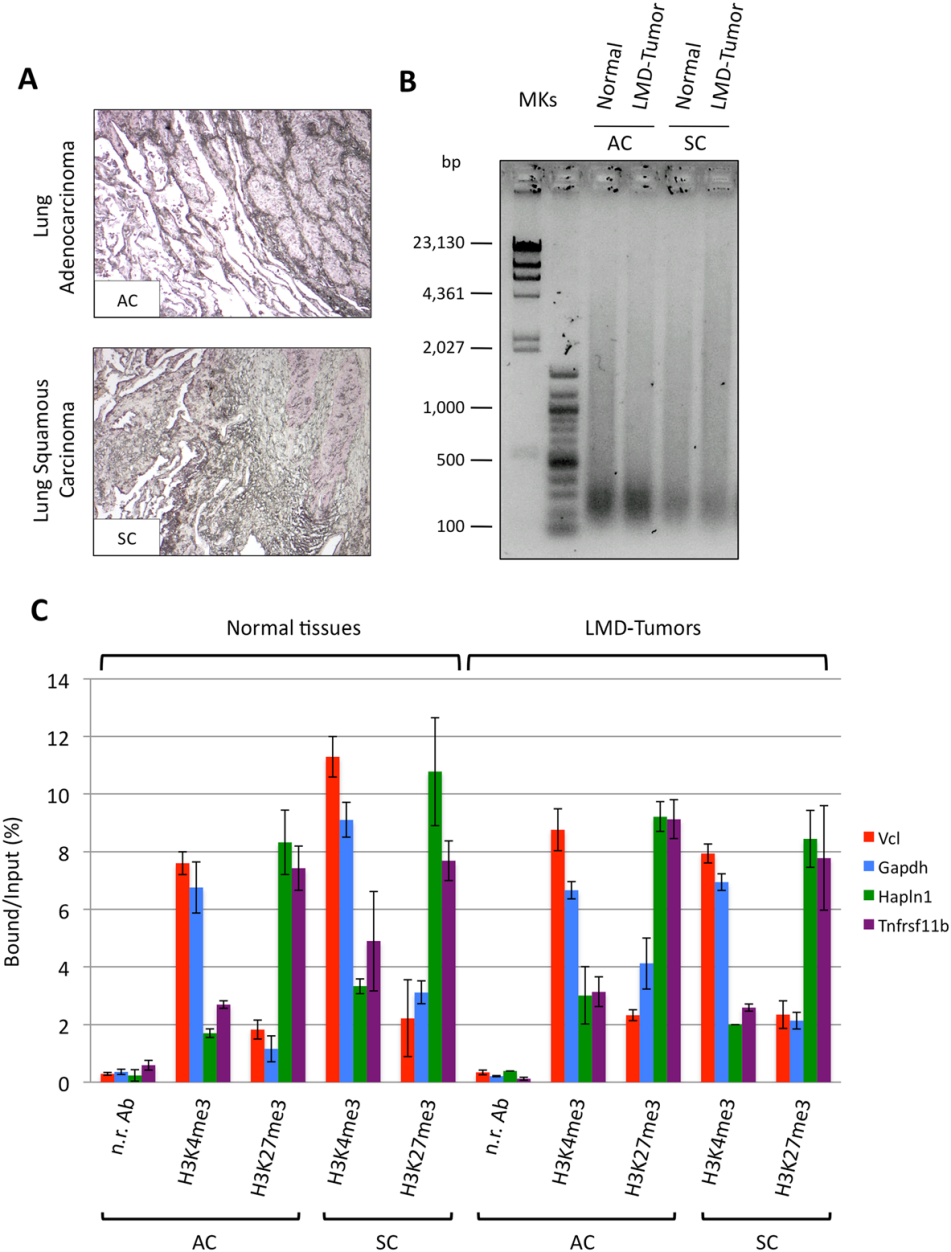
**Application of PAT-ChIP to human LMD samples.** Six tissue slides (6 μm thick) were prepared from both human FFPE lung adenocarcinoma (AC) and FFPE lung squamous carcinoma (SC) tissues and subjected to LMD to isolate normal and tumor cells. Chromatin was then immunoprecipitated with anti-H3K4me3 and anti-H3K27me3 antibodies, and the resulting purified DNA was analyzed by real-time qPCR for enrichment at specific loci. **(A)** Representative H&E staining of the two human FFPE lung samples. **(B)** Evaluation of chromatin fragmentation by electrophoretic separation on 1.3% agarose gel and SYBR® Gold staining of DNA purified only from H3K4me3 unbound fractions. **(C)** Amplification of transcriptionally active (Vcl and Gapdh) and inactive (Hapln1 and Col2a1) promoter regions by real-time qPCR (each sample amplified in triplicate). Enrichments of the promoter sequences associated with the indicated genes for H3K4me3 (n.r. Ab, non-related antibody) are expressed as the ratio between bound and input (percentage).

Finally, in order to further investigate the applicability of PAT-ChIP to LMD-FFPE samples, we performed both H3K4me3 and H3K27me3 immunoprecipitations using chromatin isolated from human lung tumor FFPE samples. Six FFPE sections (6 μm each) of either human lung adenocarcinoma or human lung squamous carcinoma samples (Figure 5A) were subjected to LMD to isolate tumor cells and normal cells. Two hundred nanograms of chromatin isolated from both either the normal or the LMD tumor components was immunoprecipitated with an anti-H3K4me3 or anti-H3K27me3 antibody (a non-related antibody ChIP was performed as control only for the lung adenocarcinoma, due to the very low amount of chromatin isolated from the lung squamous carcinoma FFPE sample; Table 5). Similar to the chromatin isolated from the mouse model, the chromatin isolated from the different components of the human tissue sections showed a homogeneous fragmentation (Figure 5B). As shown in Figure 5C (and Additional file 5), in both the human FFPE samples, the qPCR analysis showed a specific immunoselection, characterized by high levels of H3K4me3 associated with transcriptionally active genes (vinculin (Vcl), glyceraldehyde-3-phosphate dehydrogenase (Gapdh)), and H3K27me3 enrichment was observed at regulatory regions of genes expected to be non-expressed (hyaluronan and proteoglycan link protein 1 (Hapln1), tumor necrosis factor receptor superfamily member 11b (Tnfrsf11b)).

**Table 5:** Quantification of chromatin isolated from normal or LMD tumor components of human lung tumor samples

**Sample**	**Chromatin (μg)**
Lung adenocarcinoma	
Normal	0.87
LMD tumor	0.69
Lung squamous carcinoma	
Normal	0.43
LMD tumor	0.51

## Discussion

Due to the intrinsic heterogeneity of FFPE samples, potential applications of PAT-ChIP could be hampered. Therefore, we decided to investigate CNBs and LMD samples as more homogeneous sources of chromatin.

As mentioned, the thickness of CNBs required us to push the setting used for tissue fragmentation. However, the physical stress induced by sonication at higher energy levels might alter the antigenicity of chromatin proteins, causing excessive chromatin fragmentation and/or loss of the epitopes recognized by the antibody. A sign of the increased level of physical stress to which the chromatin was subjected in our experiments was the higher level of fragmentation reached with respect to control slides. Notably, the efficiency of chromatin isolation seems to increase with smaller amounts of CNB-derived samples, as demonstrated by comparing the total amount of chromatin obtained from one and four CNBs (Table 1).Interestingly, although both samples originated from the same paraffin block, the expressed genes showed an apparent lower level of H3K4me3 enrichment when the ChIP was performed using CNBs instead of tissue sections (Figure 1C). This lower enrichment could be the result of structural changes in the chromatin—due to the higher energy used for sonication (e.g., affecting the integrity of the epitopes recognized by the antibody)—which, however, do not prevent a clear discrimination between expressed and silent genes.

The use of CNBs is not devoid of limitations. The main limit consists in the impossibility of knowing, precisely, the cellular composition of the underlying tissue.

Thus, we also considered the use of LMD: unlike CNBs, LMD allows the direct separation and collection of different cell populations from the same tissue section, thus reaching higher levels of purity. LMD is now a well-established technique, and it is used in conjunction with many different downstream applications (e.g., DNA genotyping and loss of heterozygosity (LOH) analysis, DNA methylation analysis, RNA transcript profiling, cDNA library generation, proteomics discovery, and signal-pathway profiling [19]).

As a preliminary step, we verified whether the procedure could maintain its linearity in terms of total amount of extracted chromatin, and, importantly, if it produces a comparable chromatin fragmentation in function of the progressive reduction of the tissue dimensions. The investigation of this aspect was, in our opinion, of fundamental importance, since chromatin isolation could have been strongly affected by sonication performance and micrococcal nuclease digestion efficiency (used at a fixed enzyme concentration). Interestingly, we found that, at least within the range of amounts of material tested, chromatin extraction maintains an almost perfect linearity in terms of quantities of isolated chromatin. Similarly, chromatin fragmentation does not seem to be affected, indicating that the same concentration of micrococcal nuclease can be used irrespective of variations in the quantities of starting material. This last observation is very useful, since it will allow a better standardization of the entire chromatin extraction procedure, especially important when the amount of starting tissue is not quantifiable.

We thus applied the PAT-ChIP procedure to LMD lung tumor samples originating from the K-ras^v12^ transgenic mouse model using six different antibodies; notably, the LMD procedure did not impair the analysis by ChIP of the extracted chromatin, even when studying very small amounts and after histological staining. We also found that in addition to HPTMs, non-histone proteins such as Pol II and the transcription factor CTCF can be investigated in LMD samples. Most importantly, we demonstrated that the procedure can be used to investigate HPTMs in human archival samples.

Currently, a limitation of our approach to study LMD FFPE samples is that is limited to specific loci. In fact, probably due to the more extensive crosslinking procedure routinely applied to human FFPE tissues (normally fixed by using 4% of formaldehyde for a variable incubation time ranging between 16 and 48 hours), the isolation of chromatin from LMD FFPE human samples required an increased number of sonication steps. Thus, epigenomic profiling by ChIP-Seq will require further optimization of the protocol. From a translational point of view, however, the access to small quantities of patient samples for chromatin studies will allow to validate candidate loci found through other approaches (epigenomic profiling of cell lines/fresh samples, PAT-ChIP from FFPE samples, etc.).

## Conclusions

The data reported in the present work demonstrate that different cell populations from heterogeneous FFPE tissue slides when isolated by CNBs or LMD can be investigated in a more homogeneous manner by PAT-ChIP. This result adds new experimental potential to the PAT-ChIP technique and provides further tools to elucidate the role of epigenetic alterations in malignant transformation and tumor development. We believe that the diffusion of this technique will also contribute to the identification of new biomarkers and novel therapeutic strategies against cancer and other diseases.

## Methods

### Preparation of FFPE tissues from APL and lung cancer mouse models and human tumor samples

Leukemic blasts were isolated from APL transgenic mice and i.v. injected (1 × 10^6^ cells) in syngeneic recipient mice to induce secondary leukemias [22]. When a massive splenomegaly was established (usually, ≥9 days after injection), the mice were sacrificed: the spleens were collected, rapidly washed in PBS, and incubated 16 h at room temperature in 4% formaldehyde (FA) solution.

Induction of the K-ras^v12^ oncogene, in K-ras^(+/LSLG12Vgeo)^;RERTn^(ert/ert)^ expressing mice, was achieved by intra-peritoneal administration of 4-OHT (0.5 mg/injection, three times/week for 2 weeks). Lesions with a progressively more malignant phenotype (hyperplasia, adenomas, and adenocarcinomas) can be evidenced in the lungs from 4-OHT-treated mice 9 months following administration [21].

FA-fixed samples were then routinely dehydrated in a graded ethanol series (70%, 80%, 90%, and 100% - absolute ethanol) and included in paraffin through an automated tissue processor (Leica ASP300, Buffalo Grove, IL, USA) [15]. CNBs with a diameter of 0.6 mm and a width/thickness of 1 mm were produced from mouse spleens, corresponding to a volume of tissue of about 0.28 mm^3^ (which is approximately the same volume of tissue present in a spleen section with 5 mm × 5 mm × 10 μm size, used as control).

Primary human lung tumors and normal tissues were obtained from two patients who underwent surgery for therapeutic purposes at the Fondazione IRCCS Ca' Granda Ospedale Maggiore Policlinico (Milan, Italy). The patients provided informed consent, approved by the IRB of the Fondazione IRCCS Ca' Granda, and did not receive neoadjuvant chemotherapy and/or radiotherapy. The samples were fixed in formalin for no more than 24 h, dehydrated by increasing concentrations of ethanol (95% and 100%), and subsequently included in paraffin for the diagnostic procedures. The two cases were diagnosed as lung adenocarcinoma (AC) and squamous cell carcinoma (SC), respectively. The samples were used for LMD experiments as described below.

Experiments involving animals were performed in accordance with the Italian Laws (D.L.vo 116/92 and following addition), enforcing the EU 86/609 Directive (Council Directive 86/609/EEC of 24 November 1986 on the approximation of laws, regulations, and administrative provisions of the Member States regarding the protection of animals used for experimental and other scientific purposes). The mice were housed accordingly to the guidelines set out in Commission Recommendation 2007/526/EC - June 18, 2007 on guidelines for the accommodation and care of animals used for experimental and other scientific purposes. The project was notified to the Italian Ministry of Health (Project number: 21/10).

### Laser microdissection

The FFPE tissues were morphologically examined by hematoxylin/eosin (H&E) staining, and the neoplastic lesions were identified before LMD. For LMD, the FFPE samples were cut into 6-μm sections, which were immediately placed on specific LMD UV-treated glass slides with PEN membranes (Leica Microsystems) and deparaffinized by incubation in xylene (Carlo Erba, Milan, Italy) for 1 min. Tissue sections were subsequently rehydrated in decreasing concentrations of ethanol (100%, 95%, and 75%) and rinsed in deionized water for 30 s. The slides were stained with hematoxylin for 1 min, washed in deionized water, and dehydrated by incubation in 75% ethanol for 30 s. Each step was performed at room temperature. Neoplastic lesions were isolated and separated from the normal lung tissue using the LMD 6000 system (Leica Microsystems), as previously described [23]. Microdissected samples were collected into the cap of 0.2-ml microcentrifuge tubes and stored at 4°C.

### Chromatin extraction from FFPE tissues

Chromatin extraction was performed following the original PAT-ChIP protocol [15,16] with minor modifications. The FFPE tissues were first deparaffinized by sequential incubations (five times) for 10 min in 1 ml of histolemon solution (Carlo Erba) at room temperature. The samples were rehydrated by decreasing concentrations of ethanol starting from 100% through to 95%, 70%, 50%, 20%, and water (10 min at room temperature for each step). The rehydrated samples were resuspended in 0.5 ml of lysis buffer (1× Tris-buffered saline (TBS), 0.5% Tween 20, 0.1 mM phenylmethylsulfonyl fluoride (PMSF), and 10 μg/mL RNase A) and incubated for 30 min at room temperature with rotation. After centrifugation at 17,860 × *g* for 3 min at 4°C, the samples were resuspended in 0.3 ml of digestion buffer (50 mM Tris-HCl (pH 7.4), 0.32 M sucrose, 4 mM MgCl_2_, 1 mM CaCl_2_, 0.1 mM PMSF) and fragmented by sonication (3 cycles of 30 s on and 60 s off) at 40% amplitude in a −20°C thermoblock, using an EpiShear sonicator (Active Motif, Carlsbad, CA, USA). Chromatin digestion was carried out by adding 2.5 U/ml of micrococcal nuclease (N.70196Y; USB) and incubating for 1 min at 37°C. After centrifugation at 17,860 × *g* for 3 min at 4°C, the samples were then resuspended in 0.3 ml of extraction buffer (1× TBS, 0.1% sodium dodecyl sulfate (SDS)), sonicated 18 times for 5 s (10 s off) in −20°C thermoblock with an amplitude of 85% to extract chromatin, and cleared by centrifugation. The supernatant containing chromatin was collected, and the chromatin was fluorimetrically quantified by Qubit (Invitrogen, Carlsbad, CA, USA).

Chromatin was also extracted from tissue CNBs, following the same procedure, with the exception of the pre-fragmentation step, which was carried out by sonicating the samples 15 times for 15 s at 85% amplitude. The preparation of both eosin- or hematoxylin-stained lung samples and LMD samples was conducted starting directly from the rehydration (75% ethanol) instead of deparaffination step. In addition, tissue pre-fragmentation was performed by sonicating the samples 12 times for 30 s at 40% amplitude. All the other steps of the experiment were carried out following the same procedure described above, with the exception of the final sonication step that was performed by sonicating the samples 24 times for 5 s (10 s off for murine lung samples) or 48 times for 5 s (10 seconds off for human lung samples) in −20°C thermoblock with 85% amplitude.

The evaluation of the efficiency of chromatin extraction from small quantities of the starting material was conducted using the same pool of pre-fragmented tissue slides. The sonicated samples were divided, prior to nuclease digestion, into different parts (in order to obtain the amount of material of 4, 2, 1, 0.5, or 0.25 tissue slides). Nuclease digestions were performed in parallel with the same amount of micrococcal nuclease enzyme (2.5 U). The rest of the experiment was conducted following the procedure described above.

### Chromatin immunoprecipitation and DNA isolation

Chromatin was immunoselected in incubation buffer (20 mM Tris-HCl (pH 7.4), 50 mM NaCl, 5 mM Na_2_EDTA, and 0.1 mM PMSF) using 150–300 ng of chromatin for each assay (dependent on either the amount of chromatin extracted from FFPE samples in each experiment or the number of ChIP assays to perform) and incubated for 16 h at 4°C on a rotating platform with anti-H3K4me3 (39159, Lot. 01609004; Active Motif), anti-H3K9me3 (39161, Lot. 13509002; Active Motif), anti-H3K27me3 (07–449, Lot. DAM1514011; Millipore, Billerica, MA, USA), anti-H3K27Ac (ab4729, Lot. GR55451-1; Abcam, Cambridge, MA, USA), anti-polymerase II (ab5130-50, Lot. 948723, Abcam), anti-CTCF (07–729, lot. DAM1772428; Millipore), and normal rabbit IgG (Sc-2027, Lot. l2310; Santa Cruz, Dallas, TX, USA) antibody. Forty microliters of 50% *v*/*v* slurry rec-Protein G-Sepharose 4B Conjugate (pre-incubated for 16 h at 4°C with 1 mg/mL of BSA in incubation buffer; Invitrogen) was added to each ChIP assay and incubated for 3 h at 4°C. After centrifugation, the pellets were washed sequentially with 10 mL of washing buffer A (20 mM Tris-HCl (pH 7.4), 1% TritonX-100, 50 mM NaCl, 5 mM Na_2_EDTA, and 0.1 mM PMSF), 10 mL of washing buffer B (50 mM Tris-HCl (pH 7.4), 1% TritonX-100, 100 mM NaCl, 10 mM Na_2_EDTA, and 0.1 mM PMSF), and 10 mL of washing buffer C (50 mM Tris-HCl (pH 7.4), 1% TritonX-100, 150 mM NaCl, 10 mM Na_2_EDTA, and 0.1 mM PMSF). Elution was carried out by adding 220 μL of elution buffer (1× Tris-EDTA (TE)/1% SDS) and incubating for 30 min at room temperature on a rotating platform. After centrifugation, the supernatant was recovered, and the elution was repeated with 130 μL of elution buffer to obtain a final volume of 350 μL (bound fraction).

The bound fractions and previously saved inputs (5%) were decrosslinked through overnight incubation at 65°C in elution buffer/0.2 M NaCl, followed by digestion with 0.1 mg/mL proteinase K (3 h at 45°C). DNA purification was carried out using the PCR purification kit (Qiagen, Venlo, The Netherlands) following manufacturer's instructions, and the DNA was fluorimetrically quantified by Qubit (Invitrogen).

### DNA analysis

Chromatin fragmentation was checked by electrophoretic separation of DNA (decrosslinked and purified from either input chromatins or unbound fractions, as described above) on a 1.3% agarose gel. The DNA was stained alternatively with ethidium bromide or SYBR® Gold stain (Invitrogen) as a function of the quantity of DNA loaded (from 50 to 500 ng).

Purified DNA was analyzed by qPCR using the Fast Start SYBR Green Master Mix (Roche, Basel, Switzerland) and the Rotor-Gene 6000 robocycler (Corbett Life Science, Sydney, Australia). Amplifications were carried out using conditions and primer pairs described in [15,24] or reported in Table 6. The data are reported as the percentage of enrichment with respect to the input.

**Table 6:** List and sequences of primers employed for qPCR assay

**Organism**	**Gene**	**Forward primer sequence**	**Reverse primer sequence**	**bp from TSS**	
				**Start**	**End**
Mouse	Actb	5′-TTCCAGGCCCTCCCTCAT-3′	5′-GAACTTCCTGTCACAGTAGCAGGA-3′	−2,991	−2,891
Mouse	Actb	5′-GACCTCTATGCCAACACAGTGC-3′	5′-ATGGTGCTAGGAGCCAGAGC-3′	+2,548	+2,648
Mouse	Gapdh	5′-CAGATCAGCTGCCTGTGTGG-3′	5′-GAAAGTCAGCCGAGCTGCATA-3′	−2,986	−2,886
Mouse	Gapdh	5′-TCTTTCCCTTAAACAGGCCCA-3′	5′-CGTGGTTCACACCCATCACA-3′	+2,528	+2,628
Human	Vcl	5′-ATGCCAGTGTTTCATACGCG-3′	5′-CGCCCTCCTCGTGCATTAT-3′	+94	+184
Human	Gapdh	5′-TTCGCTCTCTGCTCCTCCTG-3′	5′-CCTAGCCTCCCGGGTTTCTC-3′	+95	+185
Human	Hapln1	5′-TCGGATGCTCTCAAGTTCTGC-3′	5′-TCGCCCAGAGACAAACTTAAGG-3′	+177	+267
Human	Tnsrsf11b	5′-GTGAAGGGAACAGTGCTCCG-3′	5′-GCCCGTGCTATTCTGCATTC-3′	−540	−420

## Abbreviations

4-OHT: 4-hydroxytamoxifen; AGE: Agarose gel electrophoresis; APL: Acute promyelocytic leukemia; ChIP: Chromatin immunoprecipitation; CNB: Core needle biopsy; FA: Formaldehyde; FFPE: Formalin-fixed and paraffin-embedded; H3K4me3: Histone H3 lysine 4 trimethylation; H&E: Hematoxylin and eosin; HPTM: Histone post-translational modification; LMD: Laser microdissection; LOH: Loss of heterozygosity; PAT-ChIP: Pathology tissue-chromatin immunoprecipitation; PEN: Polyethylene naphtalate; PMSF: Phenylmethanesulfonyl fluoride; qPCR: Quantitative PCR; TBS: Tris-buffered saline; TF: Transcription factor; TSS: Transcription start site.

## Competing interests

The authors declare that they have no competing interests.

## Authors' contributions

SA participated in the experimental design, carried out the ChIP experiments, and helped draft the manuscript. MB participated in the experimental design and carried out the ChIP experiments. FF carried out the *in vivo* experiments. EB participated in the experimental design of the murine models. AF selected the FFPE samples and prepared tissue LMD. SB participated in the study design and discussion. PGP participated in study design and discussion. SM conceived the study, participated in the experimental design, and helped draft the manuscript. MF conceived the study and did the experimental design and coordination, data discussion, manuscript writing, and editing. All authors read and approved the final manuscript.

## Supplementary Material

Additional file 1 **Evaluation of the applicability of PAT-ChIP to core needle biopsies (CNBs).** Amplification of transcriptionally active (Actb and Gapdh) and inactive (Hapln1 and Col2a1) promoter regions by real-time qPCR (each sample amplified in triplicate). Enrichments of the promoter sequences associated with the indicated genes for H3K4me3 are expressed as fold occupancy relative to a non-enriched region (Col2a; squared). Click here for file

Additional file 2 **Evaluation of applicability of PAT-ChIP to eosin- or hematoxylin-stained tissue slides.** Amplification of transcriptionally active (Actb, Gapdh) and inactive (Hapln1 and Col2a1) promoter regions by real-time qPCR (each sample amplified in triplicate). Enrichments of the promoter sequences associated with the indicated genes for H3K4me3 are expressed as fold occupancy relative to a non-enriched region (Col2a; squared). Click here for file

Additional file 3 **Application of PAT-ChIP to mouse LMD samples. (A)** Amplification of transcriptionally active (Actb and Gapdh) and inactive (Hapln1 and Col2a1) promoter regions by real-time qPCR. Enrichments of the promoter sequences associated with the indicated genes for H3K4me3 are expressed as fold occupancy relative to a non-enriched region (Col2a; squared). **(B)** Amplification of transcriptionally active (Actb and Gapdh) and inactive (Hapln1 and Col2a1) promoter regions—in addition to the heterochromatic major satellite sequence amplification—by real-time qPCR (each sample amplified in triplicate). Enrichments of the amplified sequences for H3K9me3 are expressed as fold occupancy relative to a non-enriched region (Actb; squared). Click here for file

Additional file 4 **Application of PAT-ChIP to mouse LMD samples. (A)** Amplification of transcriptionally active (Actb and Gapdh) and inactive (Hapln1 and Col2a1) promoter regions—in addition with the heterochromatic major satellite sequence amplification—by real-time qPCR. Enrichments of the amplified sequences for H3K4me3, H3K27me3, H3K27Ac, and Pol II are expressed as fold occupancy relative to a non-enriched region (Col2a or Actb, squared). **(B)** Amplification of two CTCF binding sites (CTCF-BS of Dnmt3a and Dnmt1 genes) and two CTCF unrelated genomic regions as controls (CTCF neg. sequences 1 and 2) by real-time qPCR (each sample amplified in triplicate). Enrichments of the amplified sequences for CTCF binding are expressed as fold occupancy relative to a non-enriched region (CTCF negative sequence 2; squared). Click here for file

Additional file 5 **Application of PAT-ChIP to human LMD samples.** Amplification of transcriptionally active (Vcl and Gapdh) and inactive (Hapln1 and Col2a1) promoter regions by real-time qPCR (each sample amplified in triplicate). Enrichments of the promoter sequences associated with the indicated genes for H3K4me3 are expressed as fold occupancy relative to a non-enriched region (Col2a or Actb, squared). Click here for file
