# Supplementary figures and images for: PAT-ChIP coupled with laser microdissection allows the study of chromatin in selected cell populations from paraffin-embedded patient samples
Source: Epigenetics Chromatin. 2014 Aug 5;7:18. doi: 10.1186/1756-8935-7-18 (PMC4124777; doi:10.1186/1756-8935-7-18)

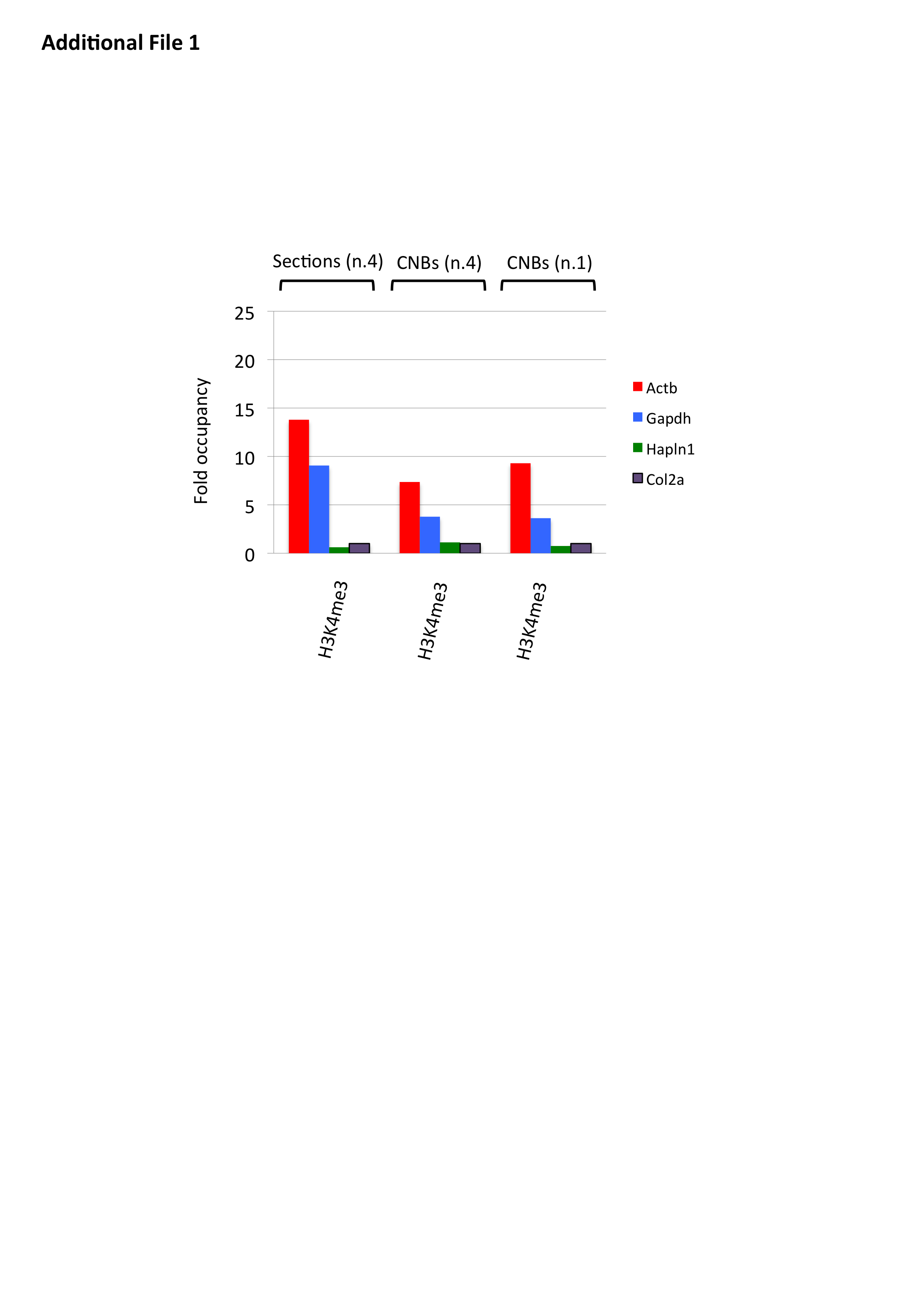

Supplement: Additional file 1 — Evaluation of the applicability of PAT-ChIP to core needle biopsies (CNBs). Amplification of transcriptionally active (Actb and Gapdh) and inactive (Hapln1 and Col2a1) promoter regions by real-time qPCR (each sample amplified in triplicate). Enrichments of the promoter sequences associated with the indicated genes for H3K4me3 are expressed as fold occupancy relative to a non-enriched region (Col2a; squared). [file 1756-8935-7-18-S1.png]

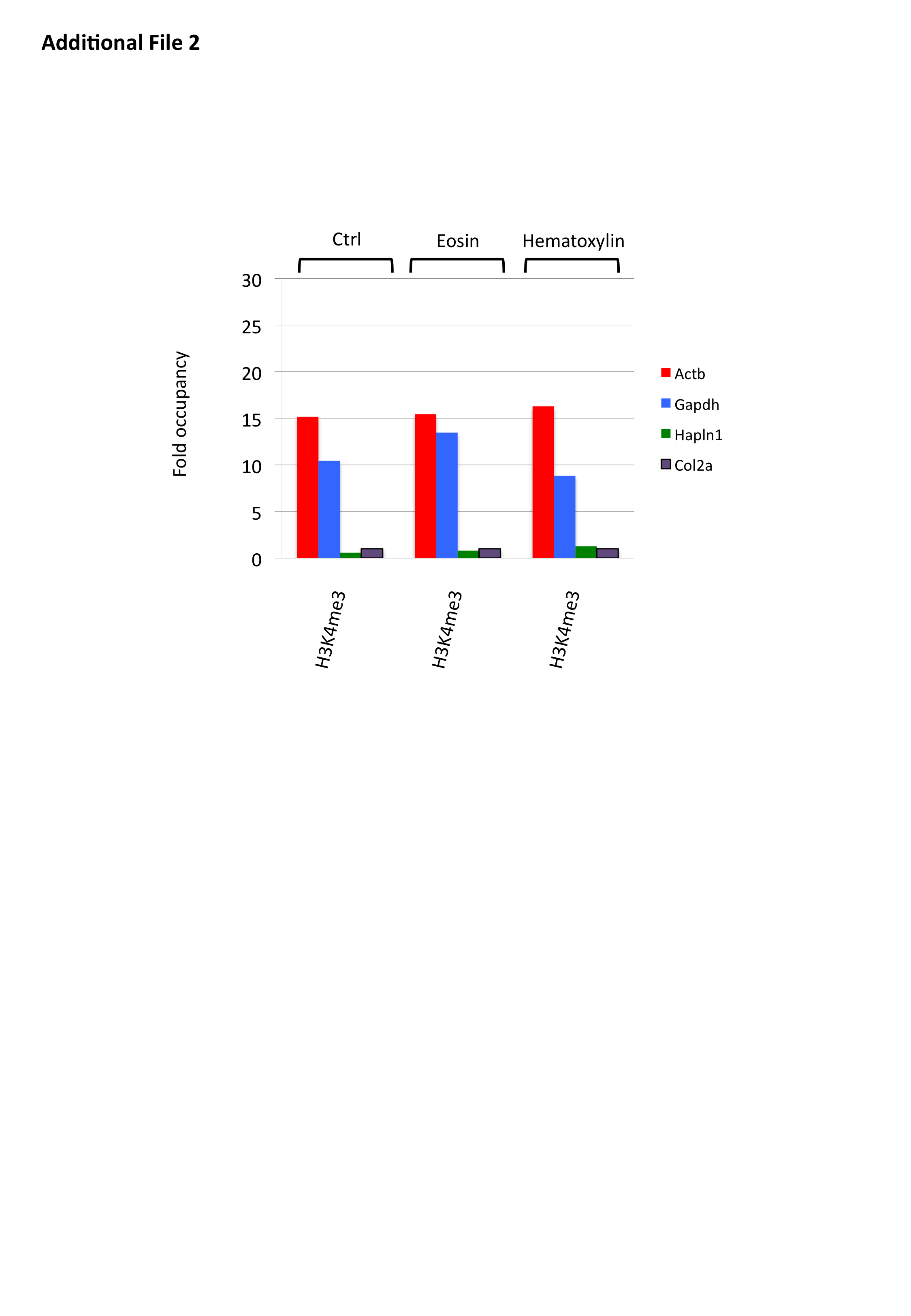

Supplement: Additional file 2 — Evaluation of applicability of PAT-ChIP to eosin- or hematoxylin-stained tissue slides. Amplification of transcriptionally active (Actb, Gapdh) and inactive (Hapln1 and Col2a1) promoter regions by real-time qPCR (each sample amplified in triplicate). Enrichments of the promoter sequences associated with the indicated genes for H3K4me3 are expressed as fold occupancy relative to a non-enriched region (Col2a; squared). [file 1756-8935-7-18-S2.png]

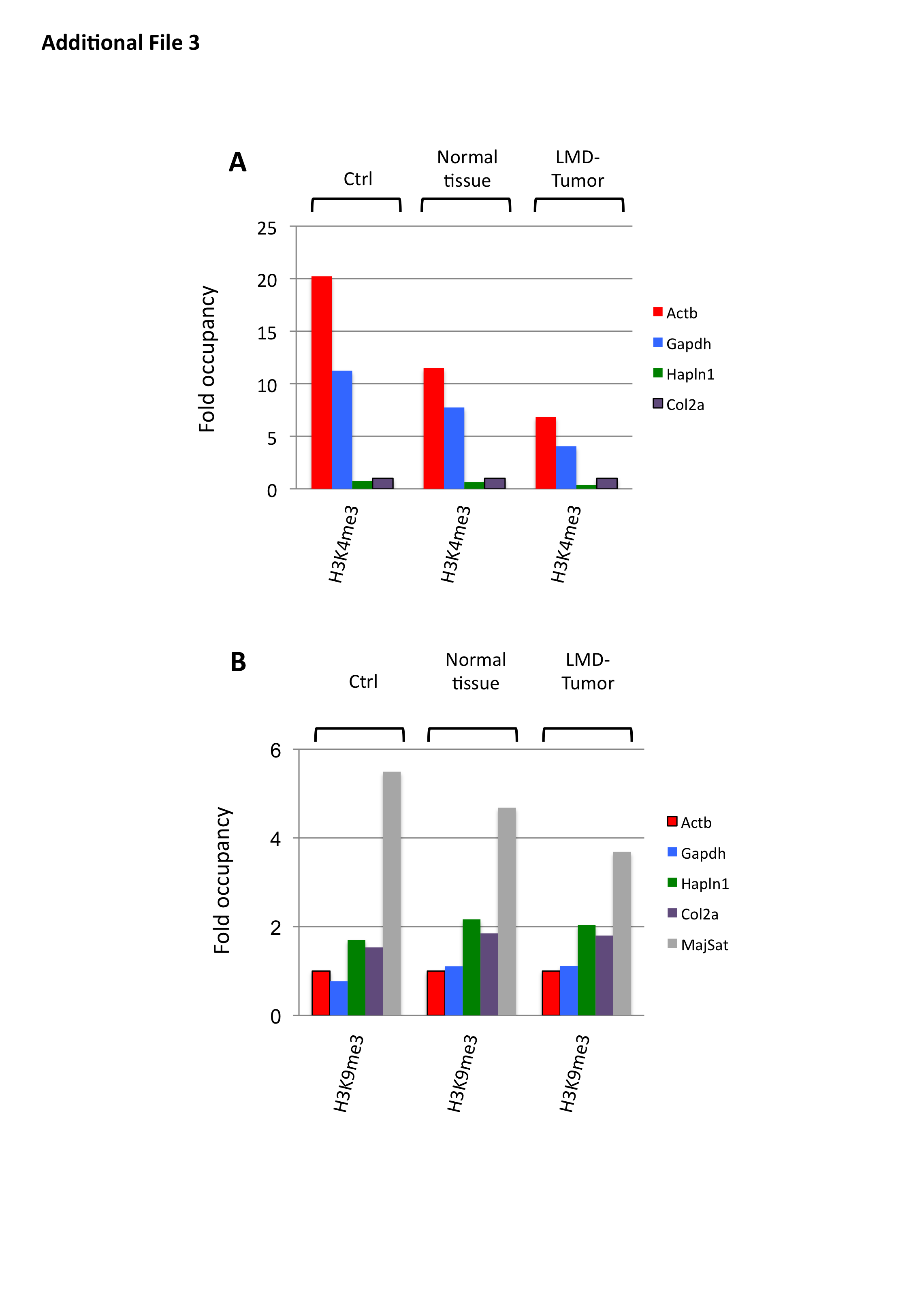

Supplement: Additional file 3 — Application of PAT-ChIP to mouse LMD samples. (A) Amplification of transcriptionally active (Actb and Gapdh) and inactive (Hapln1 and Col2a1) promoter regions by real-time qPCR. Enrichments of the promoter sequences associated with the indicated genes for H3K4me3 are expressed as fold occupancy relative to a non-enriched region (Col2a; squared). (B) Amplification of transcriptionally active (Actb and Gapdh) and inactive (Hapln1 and Col2a1) promoter regions—in addition to the heterochromatic major satellite sequence amplification—by real-time qPCR (each sample amplified in triplicate). Enrichments of the amplified sequences for H3K9me3 are expressed as fold occupancy relative to a non-enriched region (Actb; squared). [file 1756-8935-7-18-S3.png]

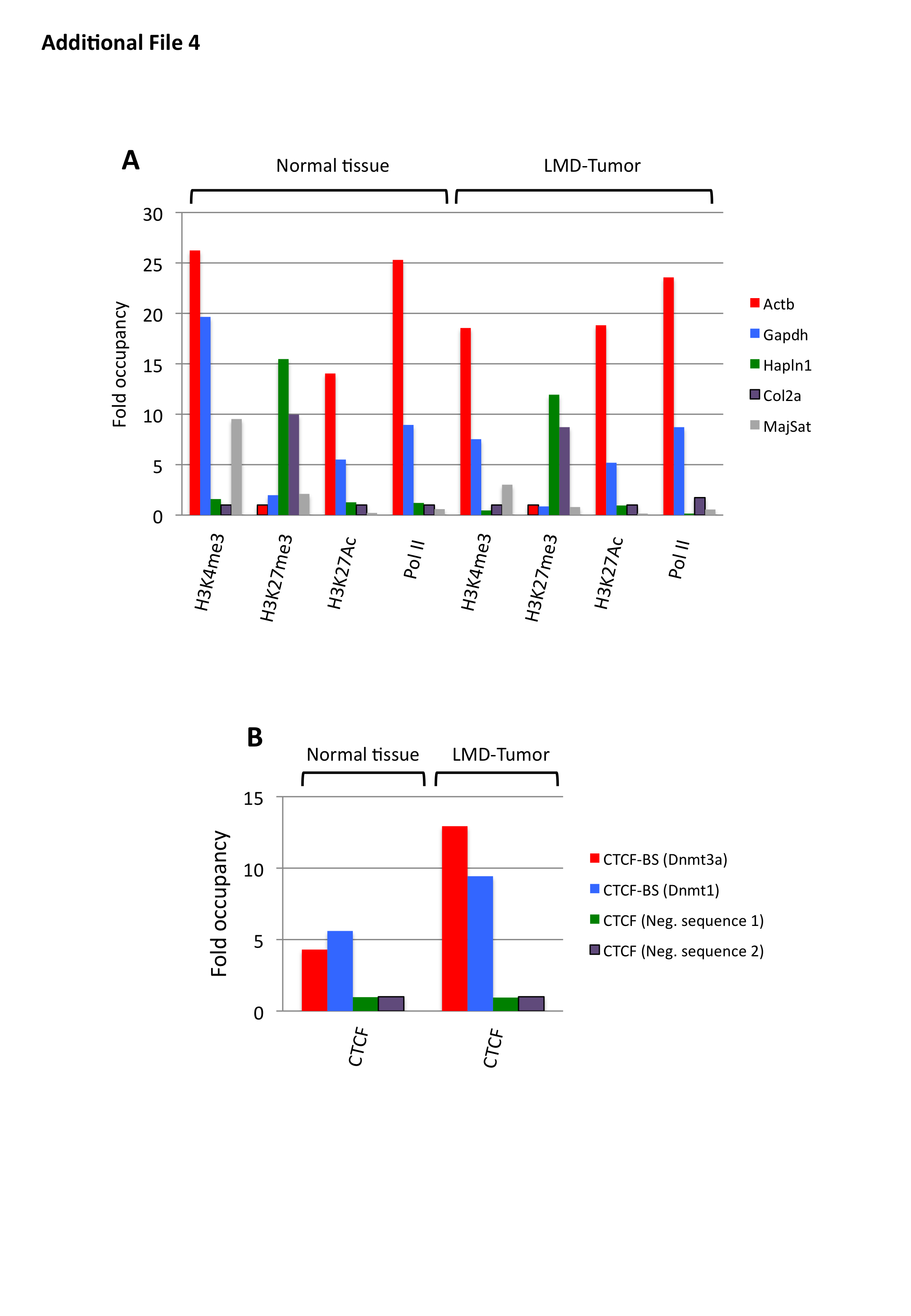

Supplement: Additional file 4 — Application of PAT-ChIP to mouse LMD samples. (A) Amplification of transcriptionally active (Actb and Gapdh) and inactive (Hapln1 and Col2a1) promoter regions—in addition with the heterochromatic major satellite sequence amplification—by real-time qPCR. Enrichments of the amplified sequences for H3K4me3, H3K27me3, H3K27Ac, and Pol II are expressed as fold occupancy relative to a non-enriched region (Col2a or Actb, squared). (B) Amplification of two CTCF binding sites (CTCF-BS of Dnmt3a and Dnmt1 genes) and two CTCF unrelated genomic regions as controls (CTCF neg. sequences 1 and 2) by real-time qPCR (each sample amplified in triplicate). Enrichments of the amplified sequences for CTCF binding are expressed as fold occupancy relative to a non-enriched region (CTCF negative sequence 2; squared). [file 1756-8935-7-18-S4.png]

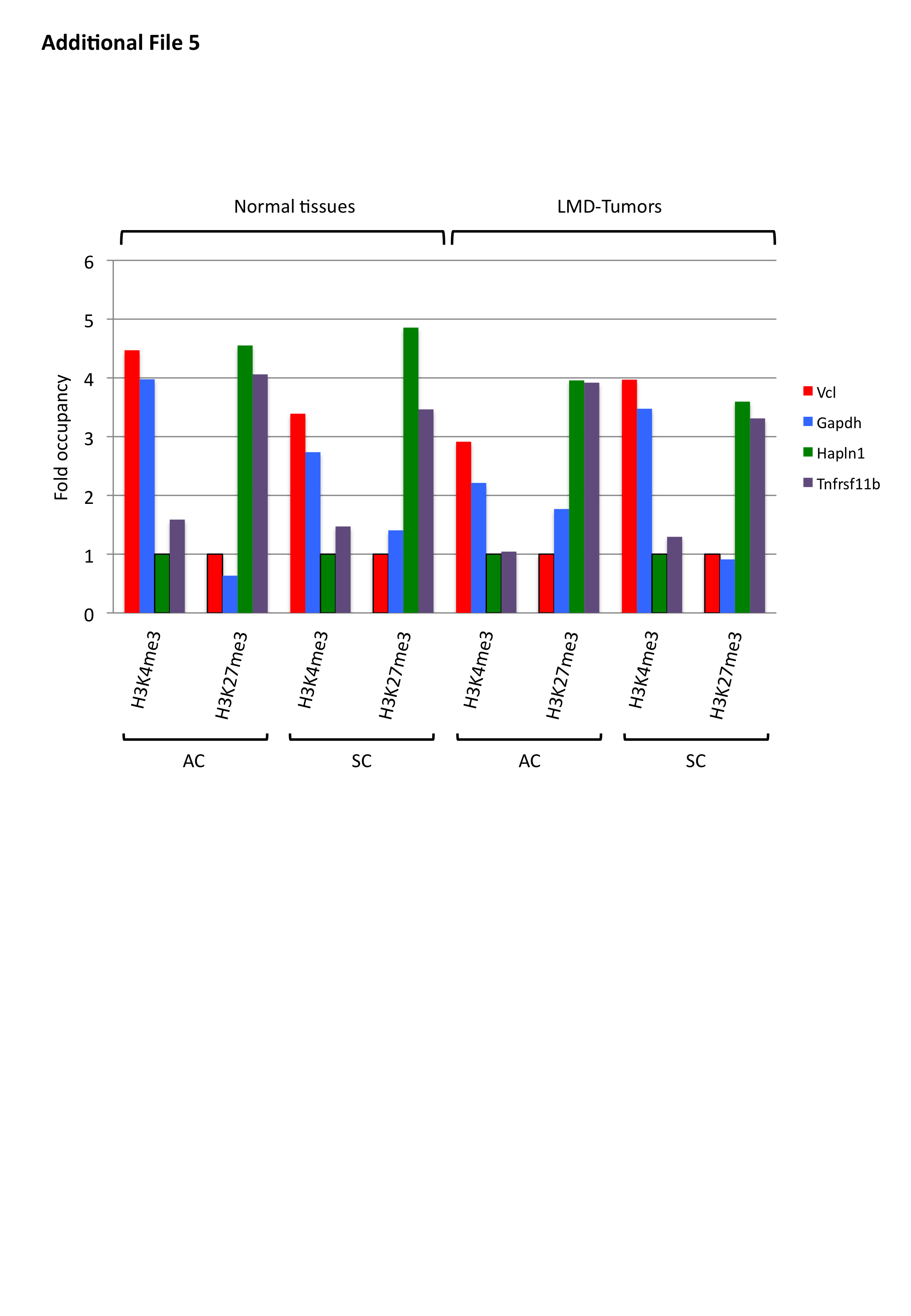

Supplement: Additional file 5 — Application of PAT-ChIP to human LMD samples. Amplification of transcriptionally active (Vcl and Gapdh) and inactive (Hapln1 and Col2a1) promoter regions by real-time qPCR (each sample amplified in triplicate). Enrichments of the promoter sequences associated with the indicated genes for H3K4me3 are expressed as fold occupancy relative to a non-enriched region (Col2a or Actb, squared). [file 1756-8935-7-18-S5.png]
